# Abuse of gabapentinoids in individuals with substance use disorders

**DOI:** 10.1111/1556-4029.70028

**Published:** 2025-03-26

**Authors:** İnci Sağlam, Rukiye Aslan, Serap Annette Akgür, Yusuf Kurtulmuş

**Affiliations:** ^1^ Manisa Mental Health and Disorders Hospital Manisa Turkey; ^2^ Institute on Drug Abuse, Toxicology and Pharmaceutical Science Ege University Izmir Turkey

**Keywords:** abuse of gabapentinoids, gabapentin, pregabalin, probation, substance use disorders, toxicological analysis

## Abstract

The abuse of gabapentinoids has increased rapidly, and death cases have been reported with increasing rates. This study examines the toxicological analysis data of substance abuse cases conducted after legal regulations were implemented in Türkiye. The focus will be on the medicolegal implications of gabapentinoid positivity in these cases. Urine samples collected between July 2021 and July 2022 from individuals (ages 18–45) presenting at Manisa Mental Health and Diseases Hospital with diagnosed or suspected substance use disorders were analyzed for the presence of pregabalin, gabapentin, and other accompanying substances. High‐resolution liquid chromatography‐mass spectrometry (Orbitrap) was employed for analysis. The analysis reports and sociodemographic data of 10,300 cases were retrieved from the patient record system and subjected to statistical analysis using SPSS 25.0. Gabapentinoid positivity was detected in 21.6% (*n* = 2224) of these cases (pregabalin (PGB) 18.9% (*n* = 1943) and gabapentin (GBP) 5.3% (*n* = 546)). Of the positive cases, 93.9% (*n* = 2089) were male, with an average age of 28 ± 6.7. Forensic cases (probation and judicial) represented the majority of admitted and positive cases 2.6% (*n* = 265) of cases tested positive for both pregabalin and gabapentin. While an overall increase in pregabalin and gabapentin positivity rates was observed over time, a statistically significant difference (*p* < 0.05) was noted between years. Seven substances were detected with gabapentinoids. Methamphetamine was the most common accompanying substance, representing nearly half of the detected substances (46.8%, *n* = 1040), followed by THC‐COOH (26.8%, *n* = 595). Despite legal regulations, the use of gabapentinoids is increasing rapidly. The study highlights the urgent need to include gabapentinoids in routine screening tests, enforce stricter prescribing guidelines, and actively prevent their illegal use.

**Highlights**

- Individuals with a history of substance use disorder seem to be at high risk for abuse of gabapentinoids.
- Pregabalin abuse and misuse are more frequent in users of pregabalin compared with gabapentin.
- Gabapentinoids are often used in combination with other substances, especially methamphetamine.
- Gabapentinoids should be included in the screening list due to their addiction potential.

## 1 INTRODUCTION

Over the past two decades, a spread of prescription and non‐prescription substances, often in combination with legal and illegal compounds—including new psychoactive substances (NPS)—have emerged for recreational use globally [1]. The internet's role in sales and distribution has fueled the rapid rise in their consumption. Alongside traditional substances like opioids and benzodiazepines, gabapentinoid abuse has also escalated significantly in the last decade [2, 3, 4]. A study examining gabapentinoid use in 65 countries/regions, representing roughly 70% of the world's population, demonstrated a substantial overall increase in consumption between 2008 and 2018 [5].

Gabapentinoids (pregabalin and gabapentin) are analgesics and antiepileptics that play a crucial role in managing chronic pain conditions that significantly impact the quality of life, such as neuropathic pain, postherpetic neuralgia, diabetic neuropathy, and fibromyalgia [6]. Over the past decade, extensive research has examined this compound's effects beyond its medical applications, investigating both its therapeutic properties (euphoria, relaxation, enhanced sociability, and dose‐dependent dissociation) and its public health implications, including substance use disorders, increasing prescription rates, and mortality risks associated with overdose [4, 7, 8, 9]. Despite precautions, widespread gabapentinoid use continues to result in numerous adverse effects, intoxications, and even deaths, as documented in pharmacovigilance databases and the literature [10, 11]. To address these concerns, regulatory bodies have taken action. In 2017, the US Food and Drug Administration designated gabapentinoids as Schedule V Controlled Substances. Subsequently, they were classified as Class C Controlled Substances in the United Kingdom. Similarly, in Türkiye, pregabalin was added to the “Green Prescription” list on April 1, 2019, requiring specialized prescriptions. Gabapentin was previously included in the list of controlled drugs requiring standard prescriptions.

In response to increasing pregabalin and gabapentin abuse in Türkiye, this study analyzes toxicology screening results from the Manisa Mental Health and Diseases Hospital Laboratory, examining cases referred for both clinical assessment and legal proceedings following recent regulatory implementation. In this study, routine drug analysis data performed on urine samples from forensic and clinical cases were retrospectively evaluated for gabapentinoids.

## 2 MATERIALS AND METHODS

This study analyzed urine samples from 10,328 individuals diagnosed with substance use disorder (SUD) who presented at Manisa Mental Health and Diseases Hospital between July 2021 and July 2022 for substance analysis. Toxicological analysis was performed using chromatographic methods (liquid chromatography – high‐resolution sequential mass spectrometry, HR‐LC–MS/MS) to screen for gabapentin and pregabalin. Samples positive for gabapentinoids were statistically analyzed in conjunction with other detected psychoactive substances. Demographic data and analysis results were obtained from the PROLIS Laboratory Operating System and Hospital Information Management System (PROBEL). Finally, the results were evaluated and interpreted within a forensic toxicology framework. Ethical approval for the study was granted by the Ege University Faculty of Medicine Ethics Committee (Ethics Committee Decision No: 21‐10.1T/23).

### 2.1 Sample preparation

Before analysis, sample integrity tests were conducted on all urine samples. Following the guidelines of the Ministry of Health Circular [12], four parameters were analyzed and evaluated spectrophotometrically, including creatinine, pH, density, and nitrite. According to these criteria, samples are classified, as shown in Table S1.

As a result of this evaluation, 28 samples were considered invalid, 449 samples were diluted, and the rest were accepted as urine samples suitable for analysis (*n* = 10,300).

A comprehensive screening was performed on each urine sample to detect a total of 147 substances, including amphetamine‐type stimulants (ATS), benzodiazepines, barbiturates, opioids, cannabinoids (THC‐COOH), cocaine and its metabolites, synthetic cannabinoids, gabapentin (GBP), pregabalin (PGB), buprenorphine (BUP) and its metabolite, lysergic acid diethylamide (LSD) and its metabolites, and venlafaxine. Samples were prepared using a “shoot and dilute” dilution technique with subsequent enzymatic hydrolysis. This process involved incubating a mixture of 500 μL urine, 530 μL distilled water, 20 μL β‐glucuronidase enzyme solution, 200 μL 32% potassium phosphate‐NaOH buffer, and 150 μL internal standard mixture (11‐Nor‐Delta9‐THC Carboxylic Acid‐D3, 6‐Monoacetylmorphine‐D3, Alprozolam‐D5, Amphetamine‐D5, Buprenorphine‐D4, Cocaine‐D3, Codeine‐D3, Diazepam‐D5, Dihydrocodeine‐D6, MDEA‐d5, MDMA D‐5, Methamphetamine D‐5, Morphine‐d3, Phenobarbital‐D5) in a deepwell plate. The mixture was transferred to a wellfilter plate and centrifuged at 3383 g for 20 min. After the centrifugation process, the supernatant is transferred to the wellfilter plate, recentrifuged again, and loaded into the analytical instrument.

Analyses were performed using a Thermo Scientific Q Exactive Focus Orbitrap LC–MS/MS system. Table 1 outlines the system features, while Table 2 provides the specific analysis parameters for PGB and GBP detection.

**TABLE 1:** HRLC‐MS/MS system features.

Parameters	Properties
Flow rate	0.3 mL/min
Run time	18 min
Injection volume	10 μL
Column	Phenomenex‐gemini 3 μm NX‐C18
Column oven temperature	40°C
Mobile Phase A	5 mM Ammonium hydrogen carbonate
Mobile Phase B	Methanol
Scanning method	Full MS dd‐ms^2^ confirmation

**TABLE 2:** Analysis features for PGB and GBP.

Parameters	Pregabalin	Gabapentin
Retention time (RT)	5 min	5.05 min
m/z (Q1)	160.13321	172.113321
Collision Energy (CE) (V)	+15 +35 +60	+15 +35 +60

### 2.2 Method validation

The method is selective for pregabalin, gabapentin, and other analyzed substances. For PGB and GBP, the calibration curve was drawn by adding the internal standard at a concentration of 150 ng/mL to the standard solutions at six different concentrations as 0, 75, 150, 300, 600, and 1200 ng/mL (*R* ^2^: 0.998). For linearity, three repetitions were performed at the same concentrations. The method is linear between 75 and 1200 ng/mL. The increased LOQ value for both parameters was accepted as 75 ng/mL.

For intra‐day and inter‐day repeatability, analysis was performed at low (75 ng/mL), medium (300 ng/mL) and high (1200 ng/mL) levels (5‐day, 5‐replicate). For pregabalin, Relative Standard Deviation (RSD%) values were determined as 1.83, 0.56, and 1.42 for intra‐day; 1.56, 0.41, and 1.06 for inter‐day, respectively. For gabapentin, it was determined as 1.76, 0.72, and 0.66 for intra‐day and 1.13, 0.40, and 0.54 for inter‐day.

In recovery studies, in 3‐day, 5‐repeat analyses, Recovery rates (in 3‐day, 5‐repeat analyses) were between 96.1% and 102.3% for pregabalin and 99.1% and 102.59% for gabapentin. For the matrix effect, samples containing 300 ng/mL standards prepared with methanol and urine samples were studied in three repetitions. No matrix effect was detected.

## 3 RESULTS

This study included 10,328 individuals (ages 18–45) diagnosed with SUD on July 1, 2022, for pregabalin and gabapentin analysis. Following the Ministry of Health criteria, 0.27% (*n* = 28) of the samples were deemed invalid (creatinine < 4.52 mg/dL and density < 1001). Case classification revealed that forensic referrals constituted 74.6% (*n* = 7704) of all cases (*n* = 10,300), encompassing Probation Services external examinations and forensic psychiatry evaluations. The remaining cases originated from clinical services—including the Alcohol and Substance Addiction Treatment Center (AMATEM) and general psychiatry clinics and emergency departments. Cases designated as “external examination” involve forensic samples analyzed from surrounding provinces and districts within the scope of probation. The emergency psychiatry outpatient clinic receives forensic and clinical cases, warranting its separate classification. Table 3 presents the sociodemographic data of the cases.

**TABLE 3:** Characteristics of the cases (*n* = 10,328) in which pregabalin and gabapentin analysis was performed.

	*n*	%
Year		
2021	4863	47.1
2022	5465	52.9
Samples	10,328	100
Invalid	28	0.27
Dilute	449	4.35
Gender		
Male	9687	93.8
Female	641	6.2
Age (Mean)	29.49 ± 7	
18–24	3034	29,4
25–34	4624	44.8
35–45	2670	25.8
Forensic	7704	74.6
Probationers	7525	72.9
Others	179	1.7
Clinical	2169	21.0
AMATEM	1752	17
Others	417	4.0
Emergency	455	4.4

Abbreviations: AMATEM, alcohol and substance addiction treatment center.

**FIGURE 1:**
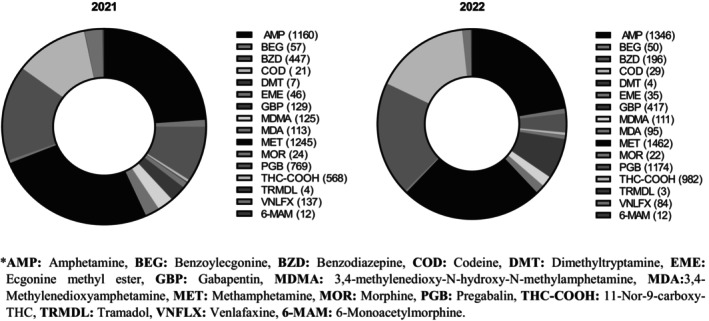
Substances detected positive in admitted cases by year. 6‐MAM, 6‐monoacetylmorphine; AMP, amphetamine; BEG, benzoylecgonine; BZD, benzodiazepine; COD, codeine; DMT, desmethyltramadol; EME, ecgonine methyl ester; GBP, gabapentin; MDA, 3,4‐Methyleneoxyamphetamine; MDMA, 3,4‐methylenedioxy‐N‐hydroxy‐N‐methylamphetamine; MET, methamphetamine; MOR, morphine; PGB, pregabalin; THC‐COOH, 11‐Nor‐9‐carboxy‐THC; TRMDL, tramadol; VNLFX, venlafaxine.

The final analysis included 10,300 specimens, comprising 9851 valid samples and 449 diluted samples, after excluding 28 invalid specimens. The inclusion of diluted samples enabled a comprehensive evaluation of all usable specimens. ATS were the most frequently detected substances, with methamphetamine at 26.3% (*n* = 2707) and amphetamine at 24.3% (*n* = 2506). PGB ranked second with 18.9% (*n* = 1943), followed by THC‐COOH at 15% (*n* = 1550) and GBP at 5.3% (*n* = 546). These numbers are the detection rates of each analyte out of the total number of cases analyzed (10,300). Of particular note, GBP detection increased at the most significant rate over time, exhibiting a 220% increase (July 1 to December 31, 2021, between January 1, 2022, and June 30, 2022). This was followed by THC‐COOH with a 72.8% increase and PGB with a 52.4% increase (Figure 1). Despite their invalid status, one sample exhibited the presence of PGB and GBP, while another contained amphetamine and methamphetamine. Among the diluted samples, 32.9% (*n* = 148) tested positive for one or more of the following analytes, which also combined with amphetamines: PGB, GBP, amphetamine, methamphetamine, THC‐COOH, BEG, MDA, MDMA, oxazepam, temazepam, clonazepam, and venlafaxine. Overall positivity rates (for all drugs) were as follows: 7.1% for invalid samples (*n* = 2), 32.9% for diluted samples (*n* = 148), and 43.1% for valid samples (*n* = 4441).

**FIGURE 2:**
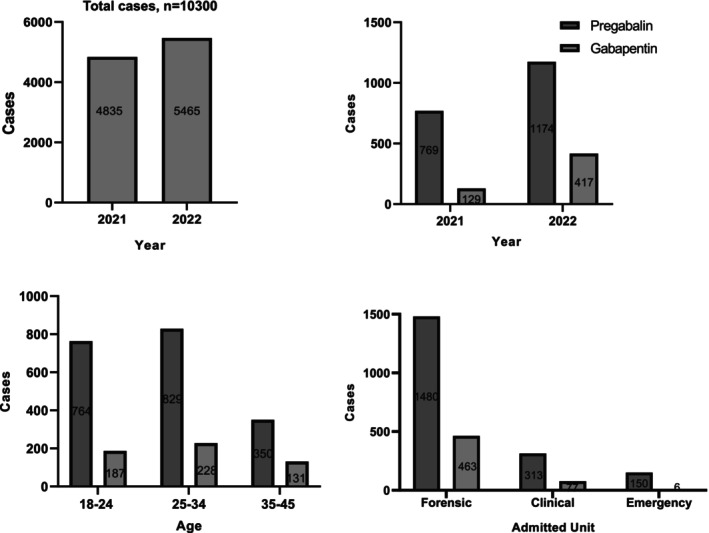
Temporal, age‐related, and unit‐wise distribution of the cases.

Gabapentinoid positivity was detected in 21.6% (*n* = 2224) of the valid cases. Of these positive cases, 93.9% (*n* = 2089) were male, with an average age of 28 ± 6.7 years. A small subset, 2.6% (*n* = 265), tested positive for both PGB and GBP. As positivity rates for both PGB and GBP increased over time, a statistically significant difference (Chi‐square, *p* < 0.05) was observed between 2021 and 2022 (Figure 2).

**FIGURE 3:**
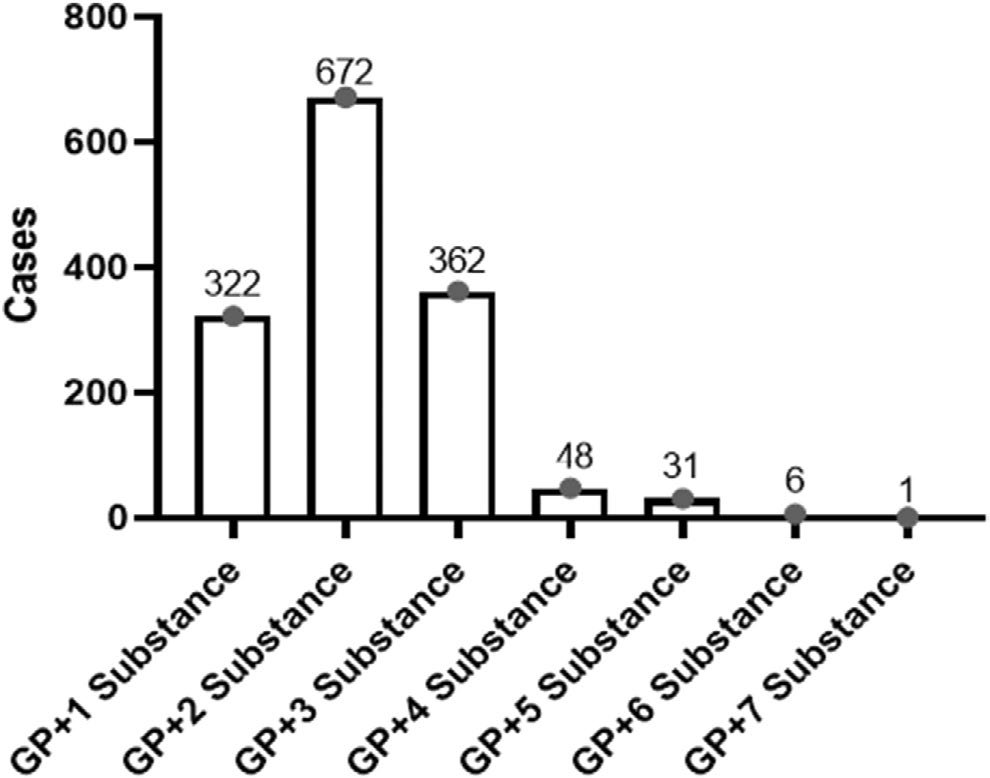
Distribution of detected poly‐substances in urine samples with gabapentinoid‐positive cases.

Among the positive cases for either PGB or GBP, 25.3% (*n* = 562) exhibited only PGB, while 6.3% (*n* = 139) had only GBP detected. Analysis revealed substance combinations alongside gabapentinoids, with up to seven substances detected concurrently (Figure 3).

Methamphetamine, an ATS, was the most common substance found in combination with gabapentinoids, representing nearly half (46.8%) of all detected substances, followed by THC‐COOH (26.8%). Table 4 provides a detailed breakdown of multiple substance combinations and individual substances detected.

**TABLE 4:** Detected substances and combinations in gabapentinoids‐positive cases.

Multiple substance combinations (*n*)	GP + 1 substance	GP + 2 substance	GP + 3 substance	GP + 4 substance	GP + 5 substance	GP + 6 substance	GP + 7 substance	Total, *N* (%)
AMP	—	574	316	46	28	6	1	971 (43.66)
BEG	5	11	15	8	8	3	1	51 (2.29)
BZD	15	6	23	14	3	4	—	65 (2.92)
COD	2	2	6	1	5	1	—	17 (0.76)
DMT	—	3	—	—	—	1	—	4 (0.17)
EME	—	7	9	7	8	3	1	35 (1.57)
MDA	—	61	34	23	19	3	1	141 (6.33)
MDMA	8	63	38	25	19	3	1	157 (7.05)
MET	54	586	320	46	28	6	1	1041 (46.80)
MOR	2	1	3	2	5	1	—	14 (0.62)
THC‐COOH	224	22	306	15	25	2	1	595 (26.75)
TRMDL	—	4	—	—	—	1	—	5 (0.22)
VNLFX	12	3	15	4	3	1	—	38 (1.70)
6‐MAM	—	1	1	1	4	1	—	8 (0.35)

Abbreviations: 6‐MAM, 6‐monoacetylmorphine; AMP, amphetamine; BEG, benzoylecgonine; BZD, benzodiazepine; COD, codeine; DMT, desmethyltramadol; EME, ecgonine methyl ester; GP, gabapentinoid; MDA, 3,4‐Methyleneoxyamphetamine; MDMA, 3,4‐methylenedioxy‐N‐methylamphetamine; MET, methamphetamine; MOR, morphine; THC‐COOH, 11‐Nor‐9‐carboxy‐THC; TRMDL, tramadol; VNLFX, venlafaxine.

## 4 DISCUSSION

The recent surge in controlled gabapentinoid usage warrants serious attention. While it is prescribed with an unapproved diagnostic code at a rate of 2/3 [13], it is used for non‐medical purposes and is widely distributed and sold on black markets. This trend has propelled the transformation of these drugs into recreational substances within the past decade [14]. The current study delves into the profiles of gabapentin and pregabalin detected in substance analyses following their designation as controlled drugs in Türkiye. We focused on cases referred to the laboratory affiliated with Manisa AMATEM, where substance use is a prevalent concern, offering a unique medicolegal perspective on the issue.

According to the General Directorate of Security (2021), the number of tablets seized by security forces in Türkiye increased by approximately 15 times from 2016 to 2020, with pregabalin‐containing drugs ranking among the top three synthetic drugs (General Directorate of Security, 2021). Aligning with these observations, 21.6% (*n* = 2224) of all analyzed cases tested positive for gabapentinoids in our study. Furthermore, the comparison of the last 6 months of 2021 and the first 6 months of 2022 reveals a concerning rise in GP positivity as PGB increased from 15.9% (*n* = 769) to 21.4% (*n* = 1174), while GBP surged from 2.7% (*n* = 129) to 7.6% (*n* = 417). This echoes data from the Forensic Medicine Institute, responsible for a significant portion of antemortem and postmortem analyses in our country, where pregabalin‐positive cases represented 3.2% of their total caseload. Also, it is worth noting that the study mentioned above focused on the years 2017–2018, prior to GPs being designated as controlled substances [15]. Nonetheless, despite the implementation of legal regulations since then, our study covering 2021–2022 reveals alarmingly high positivity rates for gabapentinoids, which underscores a critical issue warranting further attention and proactive measures. A similar surge was also observed worldwide. For instance, the longitudinal trend study published in 2023 used pharmaceutical sales data from 65 countries and regions around the world to assess global trends in gabapentinoid consumption between 2008 and 2018. The multinational average annual percentage change of gabapentinoid consumption was +17.20%, increasing from 4.17. The study showed that despite differences in healthcare systems and culture, a consistent increase in gabapentinoid consumption is observed worldwide, with high‐income countries remaining the largest consumers [7]. Regional studies provide further evidence of this alarming escalation. In France, they observed a staggering 1.233% increase in PGB consumption and a 1.185% increase in GBP in regions without social security coverage between 2016 and 2021 [16]. Data from the French addiction vigilance network also shows a sharp rise in GPs, from 3% in 2018 to 23.8% in 2019 [17]. Scotland offers another stark example, where prescribed GBP quadrupled and PGB prescriptions increased 16‐fold between 2006 and 2016 [18].

Gabapentinoids are often used in conjunction with other addictive substances. While THC‐COOH has historically been the most frequently associated substance in Türkiye [15], our study reveals a shift, with ATS now ranking first at 46.8% prevalence in this study population. Cannabis, however, remains a significant factor, coming in second place (26.7%). The widespread availability and affordability of ATS likely contribute to their prevalence in polysubstance use. The needs of users are also a factor in the combined use of methamphetamine with gabapentinoids. Among these, we can infer from our daily experiences that gabapentinoids are used to reduce some of the effects of methamphetamine use, such as loss of appetite and insomnia, and to reduce depressive and anhedonic symptoms that occur during methamphetamine withdrawal. Despite substantial international research on the combined use of opioids and benzodiazepines, studies specifically focusing on ATS and gabapentinoid polysubstance use remain limited. However, a recent study conducted in the Aegean Region of Türkiye [19] observed similar findings to those of the current study, observing the combined use of PGB and methamphetamine. While the dramatic abuse potential of gabapentinoids among individuals with a history of opioid use disorder was demonstrated by studies [2, 4, 7], our study provides further evidence for the polysubstance use of gabapentinoids and ATS. Mirroring a pattern observed in the United States and England, the “opioid crisis” led to a shift away from opioid prescriptions in favor of gabapentinoids for neuropathic pain, which resulted in a dramatic increase in PGB and GBP prescriptions, rising 150% and 350%, respectively, over a mere 5 years [13]. However, despite their affordability and accessibility, promoting the long‐term use of gabapentinoids as a replacement for opioids or in combination with them has proven insufficient in preventing severe adverse effects like psychological/physical dependence and overdose [20, 21]. The perception of safety, a scarcity of superior alternatives, and the urgent need to address chronic pain continue to drive their use. Our study revealed a low (<1%) association between opioids and gabapentinoids. The low prevalence of opioid use disorders in our study likely reflects the nature of our hospital as a psychiatric facility where medical analgesics are not prescribed. Additionally, the patient population primarily consists of individuals seeking addiction treatment at AMATEM or on probation. The co‐administration of gabapentinoids occurred more frequently with benzodiazepines than with opioids, consistent with global trends [22, 23]. The dual role of benzodiazepines as therapeutic agents at our institution complicated the differentiation between legitimate use and potential misuse.

The decrease in the age of use of GBP and PGB and the increase in the use of these substances in judicial cases such as probation, especially in order to take advantage of the gap in the legal regulation, is also an issue that needs to be emphasized. Several factors contribute to the increasing use of gabapentinoids (GPs). Despite their controlled prescription status, GPs are excluded from mandatory five‐panel screening protocols, and their unauthorized use does not constitute a criminal offense, particularly within the probation program framework. This regulatory environment allows affected individuals to maintain their occupational and social functions while potentially posing risks to themselves and others. However, it may be a challenge to distinguish between medical use and abuse. This situation can be overcome by evaluating prescriptions. Since methadone is on the routine analysis scale in some countries but not in our country, a cost‐effective approach can be provided by replacing it with gabapentinoids.

The clinical landscape presents additional challenges. Neither the DSM‐5 nor ICD‐11 includes specific diagnostic criteria for gabapentinoid use disorder, yet patients with gabapentinoid dependence—either alone or combined with other substances comprise—more than half of all SUD diagnoses in clinical practice (personal communication). This discrepancy between diagnostic frameworks and clinical reality underscores the severity of the situation. While gabapentinoids are not included in mandatory screening protocols by the Substance Abuse and Mental Health Services Administration in the United States, similar to our national policy, the European Workplace Drug Testing Society guidelines list them as secondary substances for targeted analysis.

The exploitation of gabapentinoids by individuals under judicial supervision, particularly those seeking to evade legal restrictions, presents a serious challenge. The findings indicate that individuals aged 24–35 represent a significant portion of cases (44.8%, *n* = 4624). Furthermore, alarming positivity rates for gabapentinoids were observed among those within the forensic and probation systems (75.9% for PGB and 82.6% for GBP). These results highlight a concerning pattern of gabapentinoid abuse within this population. Given these findings, we strongly advocate for the inclusion of gabapentinoids in mandatory substance screening protocols as mandated by the Ministry of Health.

## 5 CONCLUSION

This study reveals an increasing trend of gabapentinoid use alongside illicit substances like ATS and cannabis, indicating a concerning pattern of abuse despite precautions and controlled drug procedures implemented in Türkiye. Current legal frameworks appear insufficient in curbing the abuse of gabapentinoids. While these medications have legitimate medical uses as analgesics and antiepileptics, their potential for misuse places a significant burden on prescribers. The fact that these drugs are not included in routine toxicological analyses, such as probation applications, does not constitute a crime even if analyzed, and not every abuser has a history of SUD further increases this responsibility. Our findings emphasize the urgent need to include pregabalin and gabapentin in routine toxicological analysis panels and to increase awareness of their abuse potential within the judicial system.

## CONFLICT OF INTEREST STATEMENT

The authors have no conflicts of interest to declare.

## Supporting information


Table S1.
