# Supplementary material for: Abuse of gabapentinoids in individuals with substance use disorders
Source: J Forensic Sci. 2025 Mar 26;70(3):1125–32. doi: 10.1111/1556-4029.70028 (PMC12046110; doi:10.1111/1556-4029.70028)
Supplement: Supplementary file 1 — Table S1. [file JFO-70-1125-s001.docx]

TABLE S1 Urine integrity tests criteria.

| **Urine Integrity Tests**   - Creatinine - Gravity - pH - Nitrite | Creatinine <4,52 mg/dl  density < 1.001 | The sample is not urine |
| --- | --- | --- |
|  | Creatinine 4,52-20mg/dl density 1.001-1.003 | Diluted urine sample |
|  | Creatinine 20-200 mg/dl density 1.003-1.035  pH 3-11  nitrite negative | Urine sample suitable for analysis |
|  | Creatinine >200 mg/dl density >1.035  pH < 3 or >11  nitrite positive | Tampered urine sample |
